# Unassisted home births in Norway: A growing concern

**DOI:** 10.1111/aogs.15179

**Published:** 2025-06-19

**Authors:** Solveig Bjellmo, Johanne Kolvik Iversen

**Affiliations:** ^1^ Leader of Norwegian Society for Gynecology and Obstetrics Norway; ^2^ Department of Obstetrics and Gynecology Møre og Romsdal Hospital Trust Alesund Norway; ^3^ Editor of the Obstetric guidelines for the Norwegian Society for Gynecology and Obstetrics Norway; ^4^ Maternity Oslo Universitetssykehus Ullevål Oslo Norway

Norway has one of the world's lowest maternal and perinatal mortality rates. Cesarean section and operative vaginal delivery rates remain stable, even as induction rates have doubled from 2009 to 2023.^1^ These outcomes reflect a high‐quality maternity care system. Yet, a troubling trend has emerged: the number of planned unassisted home births increased from a total of 20 between 2020 and 2023 to 21 cases in 2024 alone.^2^

This raises critical questions. Why are some women opting out of a system known for its safety and comprehensive care? Reports suggest factors such as perceived lack of emotional support, previous negative experiences, and a desire for autonomy during childbirth. Some women report feeling traumatized or violated by hospital births, leading to profound mistrust in the system.^3^

A survey conducted for the Norwegian Broadcasting Corporation (NRK) found that 1 in 10 Norwegians aged 18–39 believe unassisted home birth is safe, and a further 18% were unsure.^4^ This stands in stark contrast to medical evidence: perinatal mortality is estimated to be three times higher, and maternal mortality up to 100 times higher with unassisted birth.^5^ These numbers reveal a serious information gap, one that must be addressed urgently to prevent misinformation from putting lives at risk.

Global data are sobering. According to the WHO, approximately 800 women die every day from preventable pregnancy and childbirth‐related causes, roughly one every two minutes.^6^ While these numbers primarily reflect countries without organized prenatal care and access to safe delivery facilities, they serve as a chilling reminder: childbirth is inherently risky. Norway's excellent outcomes are the result of decades of structured, evidence‐based care.

Presenting statistics is not meant to invoke fear, but to promote an evidence‐ based understanding of risks. So‐called “freebirth” activists in Norway have countered by asserting that “statistics and science is not the most important.”^7^

Healthcare professionals, including obstetricians and midwives, find this development deeply troubling. The associated risk became tragically clear in 2024, when a newborn died following an unassisted home birth in Norway. The subsequent public debate included inflammatory accusations, including labeling midwives rapists.^8^ The emotional toll on providers is significant, and there is growing concern about the future of the profession.

The debate touches on longstanding ethical dilemmas at the intersection of maternal autonomy and fetal rights. A recent commentary in *Aftenposten* by two legal experts and a pediatrician highlights the lack of legal clarity on when a fetus acquires independent rights, especially during labor.^9^ While Norwegian law permits involuntary admission of pregnant women with substance use disorders to protect the fetus, there are no equivalent legal protections during labor—although healthcare providers retain discretion to act in emergencies.^10^

In Norway, there has been public debate about whether unassisted home birth should be criminalized. For most obstetricians and gynecologists, this is neither feasible nor desirable. The current situation in the United States illustrates the dangers of punitive approaches. Women are currently prosecuted for murder following spontaneous abortions.^11^

We need a broader legal and ethical discussion about childbirth. The mother's autonomy must come first, but during labor, care providers may face situations where the mother's short‐term wish to avoid intervention conflicts with the fetus's life and the mother's likely long‐term interest in having a healthy child.

Recognizing fetal well‐being does not mean mandating hospital birth or criminalizing unassisted birth. Some groups are using “fetal protection” to push restrictions on women's rights. As professionals, we have a responsibility to push back.

Criminalization is not the answer. It undermines trust and discourages care seeking. We need dialogue. At a 2025 roundtable hosted by the Norwegian Directorate of Health, stakeholders agreed on three key priorities:

1. Rebuild midwife‐led units in hospitals
2. Develop case‐load midwifery models for relational, continuous care
3. Improve access to evidence‐based information including antenatal courses and digital health information provided by public hospitals.

These are not radical proposals. They are evidence‐based, women‐centered responses to a rising challenge. They reflect that budget cuts have eroded the humanity of our services, even if objective outcomes remain excellent. The historical underfunding of women's health must be addressed, not with lofty political promises, but with budget allocations. Unassisted home birth can no longer be dismissed as a fringe choice; it is a signal. It reflects unmet needs, eroded trust, and the failure of a system to meet the needs of those it serves. We must listen. Proactive, informed action is essential to ensure the continued safety, well‐being, and dignity of both mothers and their children.
